# Clinical manifestations and genetic analysis of 4 children with chronic granulomatous disease

**DOI:** 10.1097/MD.0000000000020599

**Published:** 2020-06-05

**Authors:** Chunyan Guo, Xing Chen, Jinrong Wang, Fengqin Liu, Yan Liang, Juan Yang, Fangfang Dai, Ning Ding

**Affiliations:** Department of Pediatric Respiratory, Provincial Hospital Affiliated to Shandong University, Jinan, China.

**Keywords:** children, chronic granulomatous disease, diagnosis, imaging, treatment

## Abstract

Pediatricians are unfamiliar with chronic granulomatous disease (CGD) because of its rarity and paucity of available data, potentially leading to misdiagnosis, late treatments, and mortality. The main purpose of this study was to summarize the clinical manifestations and auxiliary examination findings of four children with CGD confirmed by genetic testing.

This was a case series study of children hospitalized at the Pediatric Respiratory Department of Shandong Provincial Hospital. The clinical, laboratory, treatment, and prognosis data were analyzed.

All 4 children were boys. Two were brothers. The children's age was from 34 days to 3 years and 2 months at disease onset. The manifestations were repeated pulmonary infection, lymphadenitis, skin infection, and granuloma formation. Pulmonary infections were common. Abnormal responses were common after BCG vaccination. Thoracic computed tomography (CT) mainly showed nodules and masses, while the consolidation area in CT images reduced slowly. No abnormalities in cellular immune functions and immunoglobulin were found. The disease in all four children was confirmed by genetic testing. Long-term antibiotics and anti-fungal drugs were needed to prevent bacterial and fungal infections.

CGD should be considered in children with repeated severe bacterial and fungal infections. Abnormal responses after BCG vaccination and nodular or mass-shaped consolidation in thoracic CT images should hint toward CGD. Gene sequencing could provide molecular evidence for diagnosis. The treatments of CGD include the prevention and treatment of infections and complications. Immunologic reconstitution treatment is currently the only curative treatment for CGD.

## 1 Introduction

Chronic granulomatous disease (CGD) is a rare hereditary disease characterized by the congenital dysfunction of phagocytes (including neutrophils, monocytes, macrophages, and eosinophils).^[1–3]^ The mean age at diagnosis is 3.2 years for the X-linked recessive form and 11 years in patients with the autosomal recessive form.^[4,5]^ Management of CGD typically consists of prophylactic therapy and aggressive treatment of acute infections.^[1–3]^ The only curative options are hematopoietic cell transplantation and gene therapy.^[6]^

The nicotinamide adenine dinucleotide phosphate (NADPH) oxidase complex is composed of five subunits (gp91phox, p22phox, p47phox, p67phox, and p40phox) encoded by the *CYBB*, *CYBA*, *NCF1*, *NCF2*, and *NCF4* genes, respectively. CGD caused by *CYBB* gene mutation is an X-linked CGD, while CGD caused by mutations in the *CYBA*, *NCF1*, *NCF2*, and *NCF4* genes is called autosomal recessive inherited CGD. Any of those mutations will lead to a deficiency in the reduced NADPH oxidase complex in macrophages.^[1–3]^ This deficiency leads to respiratory burst dysfunction and inhibits the generation of superoxide in macrophages, consequently abrogating their capability of killing the peroxidase-positive bacteria and fungi.^[1,2]^

The major characteristic of CGD is granuloma formation induced by repeated severe bacterial and fungal infection, as well as by excessive inflammatory responses.^[1–3]^ The typical manifestations of CGD include repeated fever and local purulent inflammation such as repeated pulmonary infection, lymphadenitis, hepatapostema, osteomyelitis, dermapostasis, and cellulitis; pulmonary lesions are the most common manifestations of this disease.^[4]^

Despite its severity, pediatricians are unfamiliar with CGD because of the rarity of the disease and the paucity of available data, potentially leading to misdiagnosis, late treatments, and mortality.^[7,8]^ Therefore, the aim of the present study was to summarize the clinical manifestations and auxiliary examination findings of four children with CGD confirmed by genetic testing between 2012 and 2016. Improving pediatricians’ knowledge and awareness of the disease should improve its management.

## 2 Methods

### 2.1 Study design and patients

This was a case series study of children hospitalized at the Pediatric Respiratory Department of Shandong Provincial Hospital between October 2012 and August 2016. This study was approved by the Ethics Committee of the Shandong Provincial Hospital. All patients’ parents provided written informed consent for the publication of this case series report.

### 2.2 Data collection

The laboratory examination results, imaging findings, and genetic testing results were collected. The laboratory examinations included blood routine examinations, C-reactive protein (CRP), cellular immune function, immunoglobulin test, and pathogenic examinations. The pathogenic examinations included blood culture of bacteria and fungi, sputum culture, bronchoalveolar lavage fluid (BALF) culture of bacteria and fungi, contamination-proof fiberoptic bronchoscopic brushing assay for bacteria and fungi culture, culture of perianal abscess discharge, quantification of mycobacterial DNA in BALF, acid-fast bacillus smear, GM test of BALF, G test of serum, GM test, PPD test, and T-sport test. The imaging examinations included thoracic X-ray and CT scanning.

For the routine genetic testing, 1 to 2 ml of EDTA-anticoagulated peripheral blood were collected from the children and their parents. Genomic DNA were extracted from peripheral blood samples using standard procedures. Five candidate genes of primary immunodeficiency diseases, including *CYBB*, *CYBA*, *NCF1*, *NCF2,* and *NCF4,* were sequenced using the Illumina Nextseq500 platform (Illumina Inc., San Diego, CA). Sanger sequencing was performed to confirm the candidate pathogenic variants.

### 2.3 Follow-up

The children were routinely followed by an outpatient visit, medical record during hospitalization, and telephone. The last follow-up was on September 20th, 2019.

### 2.4 Statistical analysis

All statistical analyses were performed using SPSS 20.0 (IBM, Armonk, NY). Only descriptive statistics were used.

## 3 Results

### 3.1 Clinical characteristics

Four children, all boys, were included in this study. Their ages ranged from 34 days to 2 years and 2 months. These children were from 3 families, and patient #4 was the elder brother of patient #3. Family history was found for patient #1 (his grandfather had been diagnosed with mycotic pneumonia).

All 4 children were admitted to the hospital for symptoms of pulmonary infection (fever and/or cough). Patients #3 and #4 had a history of perianal abscesses. Patients #2 and #3 had a history of suppuration at the site of BCG vaccination. Patient #4 had a history of sepsis, purulent meningitis, and infection-related hemophagocytic syndrome (Table 1).

**Table 1:**
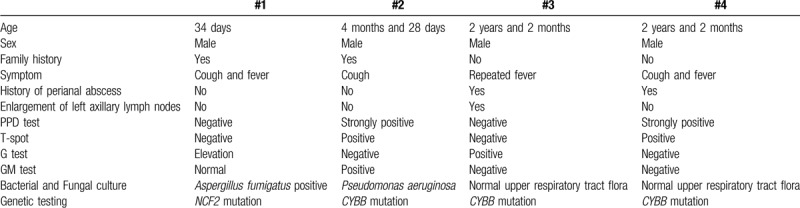
Demographic and clinical characteristics of the 4 children.

Physical examinations showed multiple enlarged lymph nodes in the left axilla of patients #2 and #3. The size of the BCG vaccination scar was about 5 × 2 cm in patient #3.

### 3.2 Laboratory examinations

For all 4 children, the blood routine examinations in the acute infection phase showed evidently increased white blood cell count, as high as 20 to 40 × 10^9^/L, mainly neutrophils. The CRP levels were 40 to 150 mg/L. No abnormalities were found in the cellular immune functions and immunoglobulin tests (Table 1).

**Figure 1:**
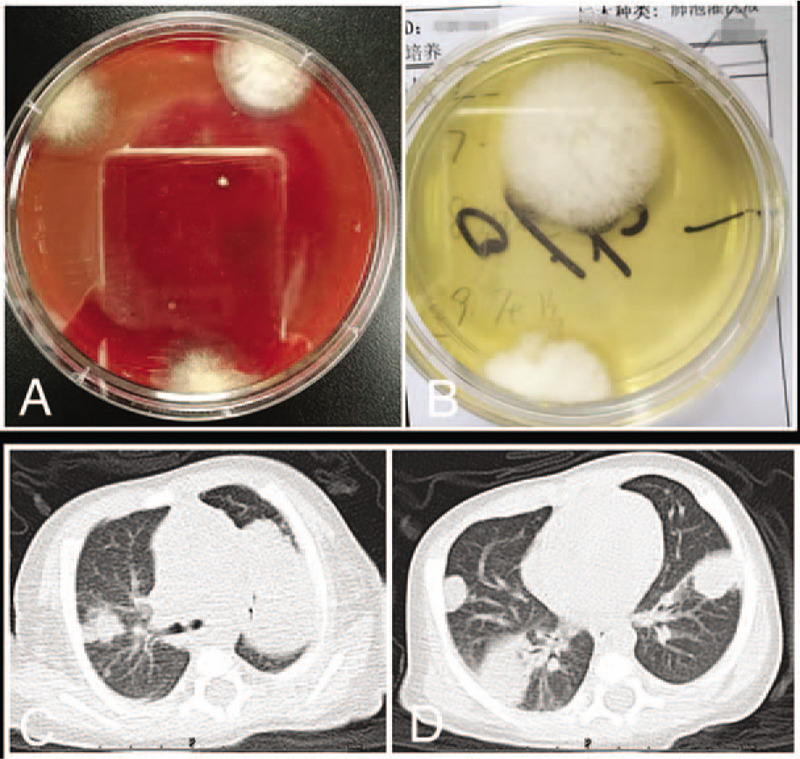
Patient #1, a 34-day-old boy admitted to the hospital for cough for 1 week, and fever for 1 day. (A) BALF culture for bacteria showed the growth of *Aspergillus fumigatus*. (B) BALF culture for fungi showed the growth of *A fumigatus*. Thoracic computed tomography at admission (C-D) showed multiple nodular high-density shadows or mass-like shadows.

For patient #1, BALF culture (Fig. 1A-B) and contamination-proof brushing assay showed infection by *Aspergillus fumigatus*. For patient #2, serum GM test results were high, the PPD test was strongly positive, the T-spot test showed positive results, and the BALF culture showed infection by *Pseudomonas aeruginosa*. For patient #3, serum G test results were high, and the PPD test was strongly positive. For patient #4, the PPD test was strongly positive, and the T-spot test showed positive results.

### 3.3 Imaging examinations

**Figure 2:**
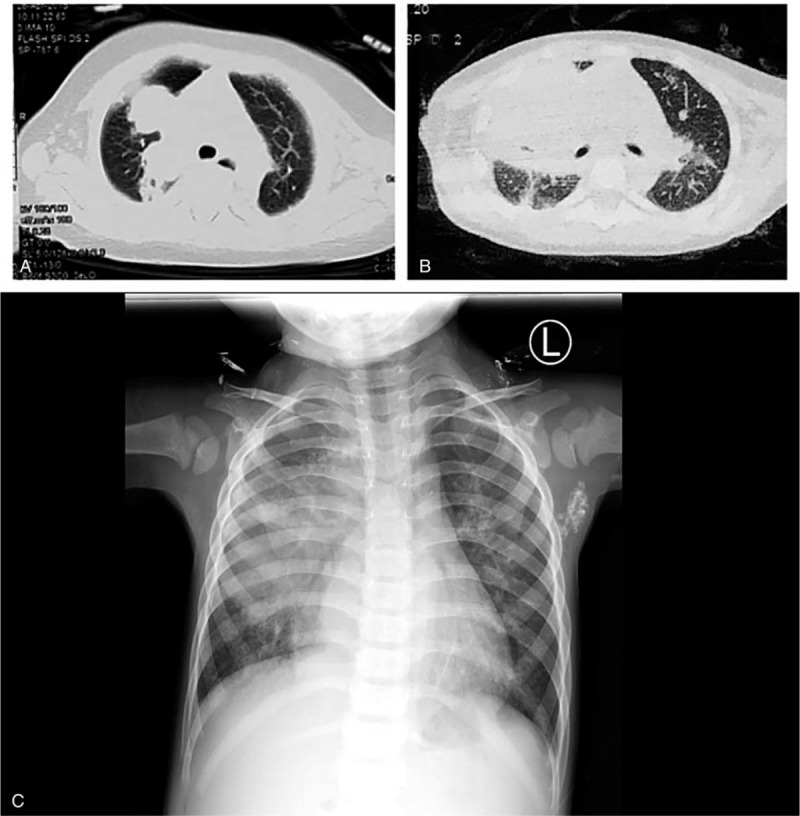
Patient #2, a boy of 4 months and 28 days, admitted to the hospital for cough for over 2 months and aggravated for 4 days. (A-B) Thoracic computed tomography showing multiple nodular high-density shadows or mass-like shadows. (C) Thoracic X-ray image 2 years later, showing calcification of the left axillary lymph nodes.

**Figure 3:**
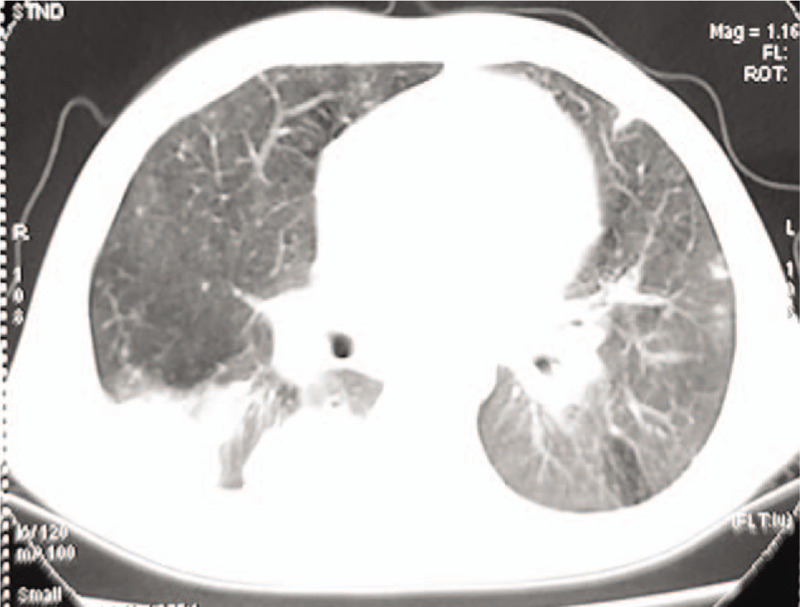
Patient #3, a boy of 2 years and 2 months, admitted to the hospital for repeated fever for over half a month. Thoracic computed tomography 7 years after disease onset, showing multiple nodular high-density shadows or mass-like shadows.

**Figure 4:**
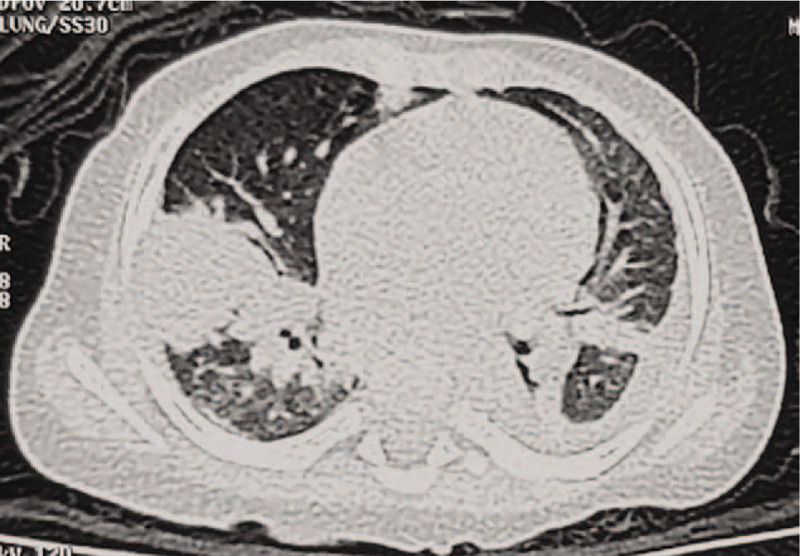
Patient #4, a boy of 2 years and 2 months who was the elder brother of patient #3, admitted to the hospital for “intermittent fever for over half a month, and cough for over 10 days.” Thoracic computed tomography 7 years after disease onset, showing multiple nodular high-density shadows or mass-like shadows.

For patients #1 (Fig. 1C-D) and #2 (Fig. 2A-B), thoracic computed tomography (CT) at disease onset showed nodular high-density shadows or mass-like shadows at the site of infection. For patient #2, the thoracic CT at disease onset showed multiple enlarged lymph nodes in the left axilla, while the chest X-ray 2 years later showed calcification of the lymph nodes in the left axilla (Fig. 2C). For patient #3, thoracic imaging at initial admission showed the manifestations of bronchopneumonia. For patient #4, thoracic CT at disease onset showed interstitial pneumonia. Thoracic CT after disease progression in patients #3 and #4 showed multiple nodular high-density shadows or mass-like shadows (Figs. 3 and 4).

### 3.4 Genetic testing

**Figure 5:**
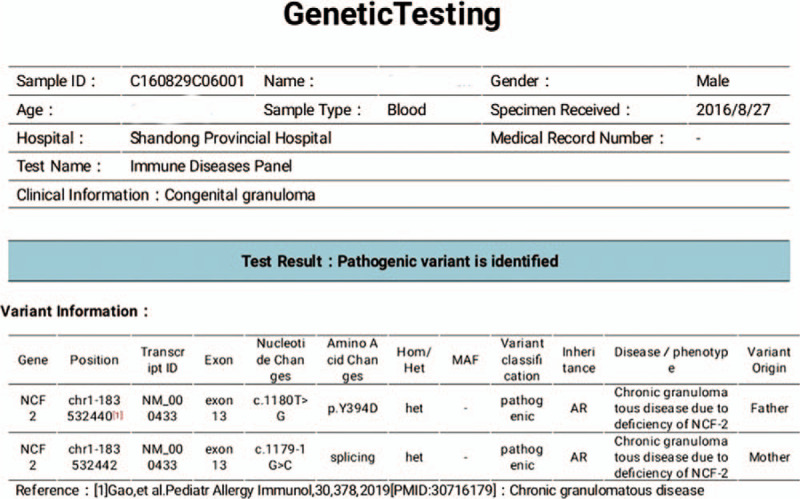
Genetic test report of Case 1 showed that the child was an autosomal recessive CGD caused by NCF2 gene mutation and both parents were carriers of the pathogenic gene.

**Figure 6:**
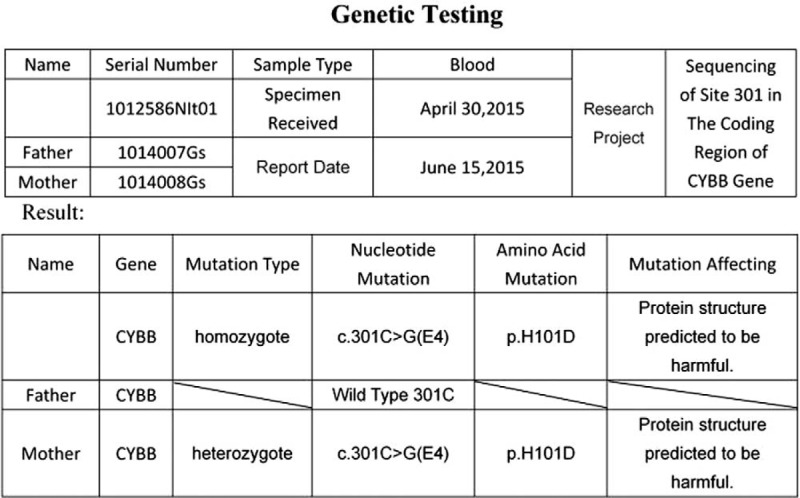
Genetic test report of Case 2 showed that the child was X-linked CGD caused by CYBB gene mutation, and the mother was the carrier of the pathogenic gene.

**Figure 7:**
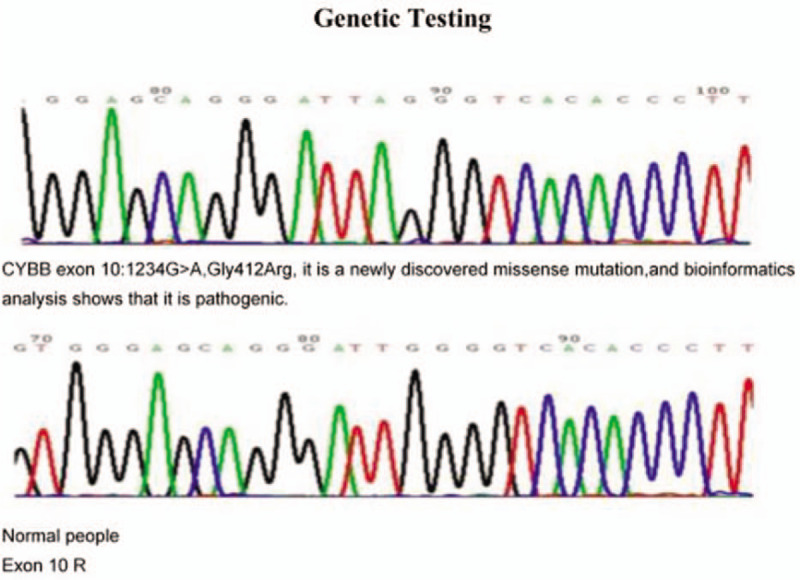
Genetic test report of Case 3 showed that the child was X-linked CGD caused by CYBB gene mutation, and the mother was the carrier of the pathogenic gene.

**Figure 8:**
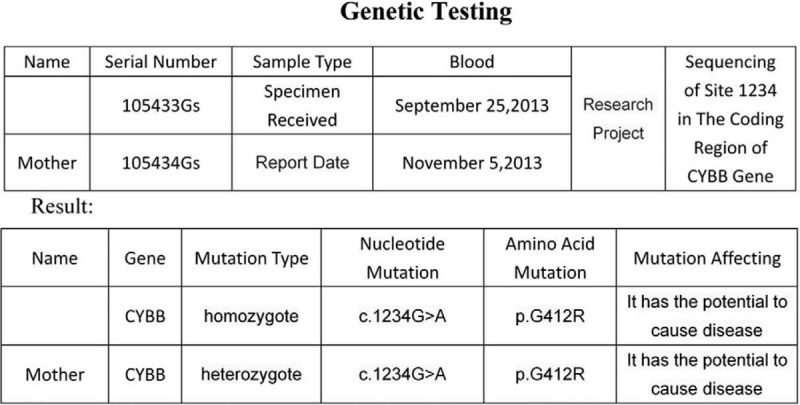
Genetic test report of Case 4 showed that the child was X-linked CGD caused by CYBB gene mutation, and the mother was the carrier of the pathogenic gene.

Patient #1 was with autosomal recessive inherited CGD with an *NCF2* gene mutation. Patients #2, #3, and #4 were with X-linked CGD with a *CYBB* gene mutation. For Case 1, both parents were carriers of the pathogenic gene. For Case 2, 3 and 4, their mothers were gene carriers (Figs. 5–8).

### 3.5 Treatments and prognosis

All 4 children received treatments with voriconazole, imipenem, and cilastatin. In addition, patient #1 also received vancomycin, while patient #2 received linezolid. After diagnosis, patient #2 received long-term treatment with cotrimoxazole and voriconazole. The last follow-up of patient #2 was on September 20, 2019, at which time his general condition was good, and the patient was on the waiting list for hematopoietic stem cell transplantation. The parents of the patients #1, #3, and #4 did not abide by the doctors’ orders to continue the long-term preventive treatment. Although they are still alive, repeated infections and progressive pulmonary lesions were found.

## 4 Discussion

Most pediatricians are unfamiliar with CGD because of its rarity and paucity of available data, potentially leading to misdiagnosis, late treatments, and mortality.^[7,8]^ The results of this study suggest that CGD should be considered in children with repeated severe bacterial and fungal infections. Abnormal responses after BCG vaccination and nodular or mass-shaped consolidation in thoracic CT images should hint toward CGD.

Abnormalities in any of the five subunits of the NADPH oxidase complex or in the G-proteins lead to dysfunctions of the oxidase complex.^[9–11]^ Most cases of CGD are X-linked recessive inherited CGD caused by mutation in the *CYBB* gene, while some cases are autosomal recessive inherited CGD caused by mutations in the *CYBA*, *NCFl*, *NCF2*, and *NCF4* genes.^[9,11]^ Over 700 mutations in the *CYBB* gene have been reported.^[12]^ In the present study, all 4 boys were confirmed with CGD by genetic testing: 3 were with gene mutations in CYBB, and one was with a mutation in *NCF2*. Their mothers were all carriers of their offspring's mutation.

The age at onset is associated with the residual activity of NADPH oxidase in macrophages.^[11,13]^ The clinical manifestations of CGD are very complex. The symptoms of CGD can appear at a very young age in serious cases or in adolescence or adulthood in relatively mild cases^[14]^ and include repeated fever and repeated pulmonary infection, lymphadenitis, hepatapostema, osteomyelitis, dermapostasis, and cellulitis.^[1–3,15]^ Pulmonary lesions are the major cause of death of this disease.^[1–4,15,16]^ All 4 children presented here were hospitalized for pneumonia, among whom 2 children had *Aspergillus* pneumonia (patients #1 and #2), while the other 2 were with *Mycobacterium tuberculosis* infection. In addition, one of the 2 children with *Mycobacterium tuberculosis* developed *Aspergillus* pneumonia during treatment for pulmonary tuberculosis. Skin or lymph node infection is relatively common in patients with this CGD.^[17]^ Two children had a history of perianal abscess, and 2 children were with skin or lymph node infection with local scar formation.

Abnormal reactions after BCG vaccination occur in children with CGD.^[17,18]^ In addition, positive results of blood culture for *Mycobacterium tuberculosis* can be found in some cases. Abnormal responses after BCG vaccination should be a cue for the diagnosis of CGD. Findings in two children included here strongly suggest that abnormal responses after BCG vaccination have a high value for the diagnosis of CGD.

In CGD, the typical granuloma manifests as non-caseous necrosis and may occur in the brain, lung, liver, spleen, and gastrointestinal tract.^[13,19]^ Generally, there is no pathogen in the granuloma, and response to glucocorticoid therapy is generally good, but complications may occur.^[20]^ Gastrointestinal tract involvement, especially inflammatory bowel disease, can be the initial manifestation of CGD.^[17]^

As pulmonary infection is the most frequent symptom of CGD, imaging of the lungs mainly shows multiple nodular high-density shadows, masses, or lobe consolidation, accompanied by nodular consolidation.^[17]^ In the present series, the initial thoracic CT images in 2 of the 4 children showed multiple nodular consolidate shadows or mass-like shadows. Therefore, imaging findings of multiple nodular consolidations have important indicative value for the diagnosis of CGD.^[1–3]^ Because of the over-activated inflammatory responses in CGD, the decrease of the consolidation area with treatment could be slower than in patients with pulmonary infections.^[21]^

CGD is a primary immunodeficiency disease due to decreased function of macrophages, but the routine cellular immune examinations and immunoglobulin examinations mainly show no evident abnormalities, and therefore, regular immunological function examinations have no indicative value for the diagnosis of this disease.^[1–3]^ Thus, the regular immunological function examinations in all four children were normal. The principle of the DHR test is that the non-fluorescent DHR can freely enter the neutrophils; when the NADPH oxidase is activated by phorbol ester, reactive oxygen species (ROS) are generated, and oxidized DHR has a green fluorescence. Therefore, children with CGD should have low or absent DHR signals.^[14]^ Of course, gene sequencing allows the characterization of the exact mutation that could cause CGD.^[14]^ In the present study, all 4 children with CGD underwent gene sequencing, which confirmed *CYBB* gene mutations in three children, and an *NCF2* gene mutation in one child.

The management of CGD mainly includes controlling the infections in their acute phase, radical treatment of the underlying diseases, and prevention of infection. For each child with CGD, aggressive pathogenic examinations and drug sensitivity tests should be conducted, the presence of *Mycobacterium tuberculosis* should be controlled for, and attention should be paid to fungal infections.^[1–3,22]^ Antibiotics should be selected according to the results of the sensitivity tests. For patients with unavailable results, empirical treatment with antibiotics should cover the oxidase-positive bacteria. For patients with empyema or abscess, surgical drainage is needed.^[1–3,17]^ For CGD children with pulmonary symptoms and/or fever from unknown causes, empirical antifungal therapy should be conducted.^[1–3]^ For patients with *Mycobacterium tuberculosis*, anti-tuberculosis drugs should be given according to the patient's condition.

Immunologic reconstitution treatment, including stem cell transplantation and gene therapy, is the only curative treatment for CGD.^[4,6,23–26]^ Nevertheless, gene therapy is still in its preliminary stage, and stem cell transplantation is still the most effective treatment method for most CGD patients. After diagnosis, transplantation should be conducted in the early life stage to protect the organ functions as much as possible.^[6]^

Long-term or even life-long trimethoprim-sulfamethoxazole should be provided for CGD patients to prevent bacterial infection before effective immunologic reconstitution treatment.^[8,27]^ The patients should avoid exposure to fungi-breeding environments or materials. Long-term or even life-long itraconazole treatment should be provided for CGD children to prevent fungal infection.^[8,27]^ The liver functions should be monitored regularly during itraconazole treatment. The use of INF-γ in treating CGD is still controversial.^[17]^

In conclusion, the understanding of CGD in pediatricians should be improved to avoid misdiagnosis and complications.^[28,29]^ CGD should be considered in children with severe bacterial or fungal infections in the lung, lymph nodes, liver, spleen, bone, or skin with an onset at a very young age, in the presence of abnormal responses after BCG vaccination, and with multiple nodular high-density shadows or mass-like shadows in thoracic CT images that disappear very slowly or not all after treatment. For CGD patients or carriers, genetic counseling and prenatal examinations should be considered. BCG vaccination should be avoided for children with abnormal results. Currently, stem cell transplantation is the most effective method for the treatment of CGD in most patients.

## Acknowledgments

We gratefully acknowledge the Shandong Medical Imaging Research Institute and the Microbiology Laboratory of Shandong Provincial Hospital for their efforts.

## Author contributions

Chunyan Guo: The main finisher of the article.

Xing Chen: Responsible for the design of the article.

Jingrong wang: Responsible for collecting imaging data.

Fengqin Liu: Responsible for collecting imaging data.

Yan Liang: Responsible for the collection of case data.

Juan Yang: Responsible for the collection of case data.

Fangfang Dai: Responsible for the follow-up of cases.

Ning Ding: Responsible for the follow-up of cases.
